# Correlates of Smoke-Free Home Policies in Shanghai, China

**DOI:** 10.1155/2014/249534

**Published:** 2014-07-01

**Authors:** Pinpin Zheng, Michelle C. Kegler, Carla J. Berg, Wenjie Fu, Jing Wang, Xilan Zhou, Dong Liu, Hua Fu

**Affiliations:** ^1^Key Laboratory of Public Health Safety, Ministry of Education, School of Public Health, Fudan University, 138 Yixueyuan Road, Shanghai 200032, China; ^2^Rollins School of Public Health, Emory University, Atlanta, GA 30322, USA; ^3^Center of Disease Control, Pudong District, Shanghai 200136, China; ^4^Center of Disease Control, Fengxian District, Shanghai 201400, China

## Abstract

*Background*. Approximately 63.7% of nonsmokers in China are exposed to secondhand smoke (SHS) in their homes. The current study documents the prevalence and correlates of smoke-free home policies in Shanghai, as well as reasons for implementing such a policy and places where smoking is most commonly allowed. *Methods*. We conducted in-person surveys of 500 participants using a multistage proportional random sampling design in an urban and suburban district. *Results*. Overall, 35.3% had a smoke-free home policy. In the logistic regression, having higher income, not having smokers in the home, having children in the home, having fewer friends/relatives who permit smoking at home, and not being a current smoker were correlates of having a smoke-free home policy (*P* < 0.05). Concern about the health impact of SHS was reportedly the most important reason for establishing a smoke-free home. Among participants with no or partial bans, the most common places where smoking was allowed included the living room (64.2%), kitchen (46.1%), and bathroom (33.8%). *Conclusions*. Smoke-free home policies were in place for a minority of households surveyed. Establishing such a policy was influenced by personal smoking behavior and social factors. These findings suggest an urgent need to promote smoke-free home policies through tobacco control programs.

## 1. Introduction

Exposure to secondhand smoke (SHS) contributes to a range of health problems among nonsmokers and children. Eliminating smoking in indoor settings can fully protect nonsmokers from the health effects of SHS [1, 2]. Having smoke-free policies in public places has positive health effects, encourages smokers to quit, and reduces cigarette consumption [3, 4]. Moreover, implementing smoke-free policies provides an opportunity to educate the public about the harm of SHS and to change social norms related to smoking, which may lead to increased adoption of voluntary smoke-free home policies in homes [2, 3, 5].

China has the most smokers and largest number of people exposed to SHS in the world. It is estimated that 63.7% of nonsmokers are exposed to SHS in their homes [6]. This widespread public health problem has persisted without any decline. According to results of China National Behavior Monitoring from 2002 to 2010 [6], there has been no reduction in the prevalence of SHS exposure over this period.

Most people spend much of their time in their homes, which continue to be a major source of SHS exposure. Despite the negative impact of SHS exposure at home in China, there have been few studies examining the prevalence or correlates of smoke-free home policies in China. In 2006, a study of an urbanized community in Shanghai showed that 26% of respondents reported a total smoke-free home policy [7]. Another study from six counties in China in 2004 indicated that only 6.3% of families completely forbade smoking at home [8]. A population survey in Guangdong Province indicated that 14.2% reported a full ban in the household [9].

Consistent with the trend of global tobacco control and the goals set by the World Health Organization (WHO) Framework Convention on Tobacco Control (FCTC), China has engaged in establishing and enforcing smoke-free policies in several Chinese cities. In March 2010, the Shanghai Public Places Smoking Control Legislation went into effect as the first provincial-level legislation on tobacco control in China. However, it is only a partial public smoke-free policy in which smoking is only prohibited in 13 types of public settings but exempted in restaurants, hotels, and other workplaces. International experience suggests that legislation mandating smoke-free public places also encourages families to make their homes smoke-free, as knowledge about the harm of SHS increases and social norms are altered [10]. In New Zealand, self-reported SHS exposure at home fell from 20% to 9% after the enforcement of a comprehensive smoke-free policy in the public places and work places [11]. Moreover, recent literature has also considered the harms of third-hand smoke (THS), which is the residual tobacco smoke contamination that remains after the cigarette is extinguished. THS may interfere negatively with household dust and air particles. In fact, previous research has demonstrated that smoking at home is linked to persistently high levels of tobacco toxins, long after active smoking has occurred in a specific setting [12, 13]. Awareness of these health effects may be related to implementation of smoke-free home policies, particularly in homes where nonsmokers and children are present. Perhaps related to these concerns, consistent predictors of smoke-free home policies have included the presence of children and of nonsmoking adults at home and having more friends and family members who smoke [14–17].

Given the gaps in the existing literature and the recent changes in tobacco control in Shanghai, the current study aimed to (1) document the prevalence of smoke-free home policies in Shanghai, (2) identify correlates of having a smoke-free home policy, (3) examine the reasons for establishing smoke-free home policies, and (4) identify locations at home where smoking is most commonly allowed. Doing so will provide a scientific basis for establishing effective interventions to reduce household SHS exposure in Shanghai and China more broadly.

## 2. Methods

### 2.1. Setting

There are seventeen districts in Shanghai: nine urban areas and eight suburban areas. Two districts were purposively sampled: Pudong New Area, located in the east of Shanghai and considered as China's financial and commercial hub with a total of 2.81 million people (characterized as urban) and Fengxian District, located in the southern part of Shanghai with a less developed economy level which ranked as the lowest among the 17 districts in Shanghai with 0.53 million residents (characterized as suburban).

### 2.2. Sampling

In each district, participants were recruited based on a random sampling design. First, 250 households were randomly selected in each district. After that the person (aged ≥18 years) whose birthday was closest to the interview date was invited in each selected household to participate.

From November 2012 to January 2013, 574 participants were invited to this study and 500 completed the questionnaire, yielding a response rate of 87%. The participants were approached by trained students from Fudan University to complete face-to-face interviews using structured questionnaires. These questionnaires were developed in English and then transformed into a Chinese version through translation and back translation. The Institutional Review Board of the Public Health School in Fudan University approved the protocol.

The sample size was calculated by estimating the prevalence of having a home smoke-free policy among the participants. Our previous study in Shanghai estimated that 26% of households had smoke-free home policies in an urbanized community [7]. It was estimated that 399 subjects were needed for each group in order to obtain a level of 5% with statistical power of 95%. However, considering the variation of prevalence of establishing home smoke-free policies among the different communities, we aimed to obtain a sample size of 500 participants.

### 2.3. Measures

Participants provided information on demographics, current household smoking policies, perceived harmfulness of SHS and THS, composition of household, social influences on smoking, reasons for establishing a smoke-free policy, and locations where smoking most commonly was allowed.

Smoke-free home policies were assessed by asking “which statement best describes the rules about smoking inside your home?” Participants were asked to select one of the following response options: “smoking is not allowed anywhere inside your home; smoking is allowed in some places or at some times; smoking is allowed anywhere inside your home; or there are no rules about smoking inside your home.” Participants reporting the first option were considered as having a complete smoke-free home policy [1]. We also asked “In what locations is smoking allowed: family/living room; kitchen; bathroom; your bedroom; other adult bedroom; child's bedroom; balconies; other.”

Reasons for establishing a smoke-free home were assessed by asking: which of 11 potential reasons were important reasons for participants to ban smoking at home. Examples of reasons included the following: to protect your family from the harmful effects of secondhand smoke and to discourage children from starting to smoke (listed in Table 2) [18]. Cronbach's alpha in the current study for the scale was 0.877. These were categorized into five topics: health, children, quitting, cleanliness, and other.

To assess perceived harm of SHS, participants were asked to indicate the extent to which they agreed with statements reflecting basic knowledge of SHS. Specifically, we asked participants to indicate their level of agreement on a scale of 1 = strongly disagree to 4 = strongly agree with the following three items: “breathing smoke from other people's cigarettes causes heart disease in adults”; “inhaling smoke from someone else's cigarettes can cause lung cancer in nonsmokers”; and “inhaling smoke from someone else's cigarettes can harm the health of babies and children” [19]. In addition, participants were asked about their beliefs related to the harm of THS using the same response options. This newly developed scale included the following five items: “being in a room where others previously smoked has no impact on your health”; “as soon as people stop smoking in a room, the room no longer has any trace of dangerous particles present”; “after someone smokes in a room, dangerous particles are left behind in the dust, air, and surfaces in the room”; “there are no health risks associated with being in a room where someone previously smoked”; and “dangerous particles from smoking can remain in a room for days or weeks.” Cronbach's alpha for the SHS items, THS items, and all 8 items was 0.85, 0.76, and 0.84, respectively.

Influence of other household members, friends, and relatives on having a smoke-free home policy was measured by respondents' answers to the questions: “how many of your friends and relatives are smokers?” and “how many of your friends and relatives allow smoking in their home?” with response options of “all, most, about half, less than half, a few, or none?” The smoking status of household members was assessed using two questions: “including yourself, how many smokers live in your home?” and “does your spouse or partner currently smoke cigarettes?” We also asked about the number of children at home under the age of 18 years and under the age of 5 years.

Smoking status and history were also assessed. Ever smokers were defined as those who had smoked at least 100 cigarettes in their lifetime [20, 21], and current smokers were defined as those reporting smoking in the past 30 days. The questionnaires also covered smoking related information on average daily cigarette consumption in the past week, age at smoking initiation, and number of previous quitting attempts.

### 2.4. Statistical Analyses

Fisher's exact tests or *χ* ^2^ tests were used to examine group differences among those with and without complete smoke-free policies for categorical variables, and the Student* t*-test was used to examine differences between groups for continuous variables. Binary logistic regression was used to investigate correlates of having a complete smoke-free home policy. Specifically, all variables that were associated with policy status in bivariate analyses at *P* < 0.10 were entered into the model using forced entry. These variables included gender, average personal income, setting, having a smoker at home, having child(ren) less than 5 years old, number of friends who smoke, number of friends who permit smoking at home, and current smoking status. Alpha was set at 0.05 and SPSS 21.0 was used to conduct the analyses.

## 3. Results

Table 1 presents participants characteristics and bivariate analyses. Males accounted for 48.2% of the total sample. Participants aged 20–39 years accounted for 45.4%. Overall, 29.0% were current smokers (58.1% among men and 1.9% among women). In our sample, 61.6% of participants had smokers at home. In all, 176 participants (35.2%) had smoke-free home policies. Bivariate results indicated that those with a complete smoke-free home policy were more likely to be female (*P* = 0.02), living in an urban area (*P* = 0.049), and with no smokers at home (*P* < 0.001). In addition, children under the age of 5 years at home (*P* = 0.013), fewer friends who smoke (*P* < 0.01), fewer friends who allow smoking at home (*P* < 0.001), and being a nonsmoker or ex-smoker were associated with having a smoke-free policy (*P* < 0.001).

Table 2 shows the final logistic regression model indicating significant correlates of having a complete smoke-free home policy. Having children less than 5 years old at home was positively associated with the presence of a smoke-free policy (*P* = 0.007). Having smokers at home (*P* < 0.001) and having more friends or relatives who allow smoking at home (*P* < 0.001) were negatively associated with having a complete smoke-free home policy. However, gender, area, and number of friends/relatives who smoke were not significantly associated with the presence of a smoke-free policy.

Health concern was the most important reason for establishing a smoke-free home (2.74 ± 0.64), followed by “other” concerns and concerns about cleanliness (2.38 ± 0.91 and 2.37 ± 0.92, resp.; see Table 3). Participants with complete smoke-free home policies had higher scores related to health concerns, children-related concerns, cleanliness concerns, and “other” concerns, compared to those without a smoke-free policy.

For the participants with no or a partial ban, the most common places where smoking was allowed included the living room (64.2%), the kitchen (46.1%), and the bathroom (33.8%; see Table 4). Significant differences in locations where smoking was allowed were detected between those with a partial ban and those without any restriction. For the participants without any smoke-free policy, 28.1% allowed smoking in a child's room.

## 4. Discussion

Smoke-free home policies may reflect the social norms related to smoking and attitudes about smoking and SHS in environments in which there are no widespread public campaigns promoting smoke-free homes [22]. There is also consistent evidence that smoke-free home policies not only reduce exposure to SHS but also increase cessation rates and decrease cigarette consumption in adult smokers [23]. Also, smoke-free home policies may create and reinforce life-long antismoking behavioral values and norms among youth. The association between having smoke-free home policies and reduced adolescent smoking behaviors has been confirmed in various studies [23, 24]. Given the need to understand the prevalence and correlates of having smoke-free home policies, the current study examined these phenomena among urban and suburban residents in China. Sociodemographics, household composition, social influences, and individual smoking behavior were major factors associated with policy status. In addition, we documented the most important reasons for creating these policies and the places at home most commonly exempt from any rules about smoking.

The current study showed that 35.3% participants had total smoke-free home policies, with 39.4% having them in the New Pudong Area and 30.8% in Fengxian District. These results indicate higher rates than in prior studies in which rates of 6.3%, 14.2%, and 26% were documented in 2006, 2010, and 2009 [7–9]. This may reflect differences in location, such that smoke-free home policies are more common in Shanghai than in other areas in China or may reflect an overall increasing prevalence of smoke-free homes since these earlier studies. The passage of smoke-free policies in the workplaces and public places may be resulting in a shift in social norms and ultimately in more voluntary smoke-free homes [22]. The establishment of smoking control legislation in public places in Shanghai, which had a positive influence on broad social norm changes [25], may account in part for the increased prevalence of smoke-free homes documented here. However, because Shanghai only implemented a partial smoke-free public policy with limited enforcement, positive change on social norms and behavior is not as obvious as in other countries with 100% smoke-free policies. Given the fact that a higher proportion of Chinese adults are exposed to SHS at home than in most other low- and middle-income countries with a high burden of tobacco use [26], there is considerable work yet to be done to promote voluntary smoke-free policies in private spaces in China.

The results of this study showed that social factors were significant correlates of having a smoke-free home policy. Having children less than 5 years of age was an important factor. The one child family planning policy in China promotes the value and status of children at home, which is also a good opportunity to advocate for having a smoke-free home policy in families with children. However, having children under 18 years old was not associated with having a complete smoke-free home policy, which is consistent with other studies [27, 28]. People tend to believe that older children may not be sensitive to SHS. Educational outreach should grasp these opportunities and focus on the information that SHS is dangerous to all nonsmokers of all ages, including older children and adolescents.

Having smokers at home was also an important factor related to establishing a smoke-free home. The realities of smoke-free home policies are different in families with smokers or without smokers. For the nonsmoking family, these policies are mainly established for visitors; for the smoking family, these policies are set up for both residents and visitors. As presented in other studies, participants without smokers at home report higher prevalence of smoke-free policies compared to those with household members that smoke [22]. Smokers who live in a smoke-free home have been shown to be more likely to have made a quitting attempt and maintain abstinence compared to smokers without a smoke-free home, indicating that smoke-free home policies act as a part of effective cessation support systems. For families without smokers, establishing a smoke-free home policy is also important not only to protect the health of families, but also to reduce the risk for adolescents initiating smoking. Studies suggested that the lack of smoke-free home policies, even in homes without smoking parents, may weaken communication of parental antismoking values [29]. Therefore, the concept of a smoke-free policy should be advocated in all households, regardless of household composition and smoking status. Moreover, we documented a negative association between the perceived proportion of relatives or friends who allow smoking at home and having a smoke-free home. This suggests that smoking bans among their friends and relatives may also work as an interpersonal stimulus or reinforcement to establish household smoking bans.

Health concern was the most important reason for implementing a smoke-free policy, indicating an understanding of the health effects of SHS and potentially THS. Still some people allowed smoking in certain rooms instead of going totally smoke-free. Roughly 64% of participants reported that smoking was allowed in the living room and roughly 45% reported smoking was allowed in the kitchen. Studies have confirmed that a complete smoke-free home versus a home with some level of restrictions is more effective in reducing the likelihood of adolescents smoking and increasing the likelihood of quitting attempts among the smokers [22–24]. The fact that the current Shanghai smoking control legislation permits smoking rooms in some public places may contribute to a misconception among individuals that these partial restrictions are sufficient.

Compared to participants with low average income (less than 1000 RMB per month), participants with middle average income (1001–3000 RMB per month) were less likely to report smoke-free home policies. Similar association was found in other studies [15, 16, 28]. The importance of establishing a smoke-free home has not been completely understood by the general public in China based on our findings indicating where smoking is most commonly allowed among those without a smoke-free home.

It is clearly stated in “the Twelfth Five-Year Plan for Economic and Social Development of China” that smoking should be prohibited in public places [30]. International experience in tobacco control showed that increases in smoke-free homes often follow smoke-free workplace and public place legislation; therefore, it is expected that there would be more households going smoke-free in the future in China as tobacco control is strengthened. Establishing smoke-free home policies is not a separate part of tobacco control, rather, it should be integrated into a comprehensive tobacco control strategy.

Several limitations in this study need to be addressed. This survey relied on self-report by participants, which may lead to some recall and social desirability bias. As a cross-sectional study, when exploring the factors associated with having a smoke-free home policy, there is uncertainty regarding the temporal sequence between establishing a smoke-free home and social factors related to rules about smoking at home. In addition, information about smoking behaviors and quitting intentions of smokers in the household were not collected if the respondent was not a smoker. Also, our sample size of 500 participants, while reasonably large and well powered, does not allow us to further stratify analyses. Furthermore, there may be some significant difference in attitudes towards smoke-free home policies between those who agreed to participate in this study and those who refused to participate. Despite the limitations, this study provides empirical evidence for the need to promote smoke-free home policies in Shanghai and, more generally, in China.

## 5. Conclusions

In conclusion, this study documented the prevalence of smoke-free home policies and factors associated with having such a policy in Shanghai. In addition, it examined the most common reasons for going smoke-free and documented places most commonly exempted from any restrictions at home. These findings have important implications for informing tobacco control efforts aimed at decreasing SHS exposure and altering social norms regarding smoking in China.

## Figures and Tables

**Table 1:** Participant characteristics and bivariate analyses comparing those with a complete smoke-free home policy with those without a complete policy.

Characteristics	Total *N* = 500	Complete policy *n* = 176	No or partial policy *n* = 324	*P *
*Sociodemographics *				
Gender				
Male	48.2	40.9	52.2	0.021
Female	51.8	59.1	47.8	
Ethnic				
Han	99.6	99.4	99.7	0.192
Others	0.4	0.6	0.3	
Age				
<20	0.6	0.6	0.6	
20–29	25.0	26.1	24.4	
30–39	20.4	17.6	21.9	0.941
40–49	19.6	20.5	19.1	
50–59	18.2	18.8	17.9	
≥60	16.2	16.5	16.0	
Education				
Less than high school	51.5	46.3	54.4	
High school graduate	19.2	18.9	19.4	0.173
Some college/vo-tech	13.1	14.3	12.5	
College graduate or higher	16.2	20.6	13.8	
Work status				
Employed full-time	59.5	57.9	60.5	
Employed part-time	9.9	8.2	10.7	0.925
Retired	19.0	21.1	17.9	
Homemaker	7.5	7.6	7.5	
Average personal income				
Less than 1000 Yuan	27.1	35.5	30.0	
1001–2000 Yuan	26.4	20.5	24.4	0.092
2001–3000 Yuan	29.6	23.5	27.5	
More than 3000 Yuan	16.9	20.5	18.7	
Marital status				
Married	86.6	87.5	86.1	0.750
Single	10.4	9.7	10.8	
Others	3.0	2.8	3.1	
Setting				
Urban	49.9	39.4	60.6	0.049
Suburban	50.1	30.8	69.2	
*Knowledge about smoking *				
Knowledge about SHS	9.5 ± 1.7	9.6 ± 2.2	9.4 ± 1.6	0.172
Knowledge about THS	9.1 ± 1.7	9.2 ± 2.0	9.1 ± 1.5	0.550
*Social factors *				
Have a smoker at home	61.6	43.8	71.3	<0.001
Average smokers at home	0.8 ± 0.8	0.9 ± 0.7	0.6 ± 0.7	<0.001
Have child(ren) under 18 years old	46.8	49.7	45.2	0.353
Have child(ren) under 5 years old	25.5	32.2	21.9	0.013
Number of friends/relatives who smoke				
≥half	47.6	36.6	53.6	<0.001
<half	52.4	63.7	46.4	
Number of friends/relatives who permit smoking at home				
≥half	45.8	28.5	54.3	<0.001
<half	54.2	71.5	45.7	
*Smoking status *				
Current smokers	29.0	17.0	35.5	
Nonsmokers	68.0	81.7	61.1	<0.001
Ex-smokers	3.0	2.3	3.4	
*Among smokers *				
Number of days of smoking, past 30 days	23.6 ± 10.8	21.2 ± 11.2	24.3 ± 10.4	0.133
Number of quitting attempts, past 12 months	1.9 ± 4.0	3.0 ± 7.4	1.6 ± 2.4	0.080

**Table 2:** Multiple logistic regression model indicating correlates of having a complete smoke-free home policy.

Predictors	Adjusted OR	95% CI
Gender (female versus male)	0.74	0.43–1.26
Average personal income (ref: <1000 RMB per month)		
1001–2000	0.46	0.35–0.98
2001–3000	0.68	0.35–0.96
More than 3000	0.92	0.53–1.59
Setting (suburb versus urban)	0.69	0.44–1.11
Have a smoker in home	0.37	0.23–0.60
Have child(ren) less than 5 years old	2.04	1.26–3.29
Number of friends/relatives who smoke (<half versus ≥half)	1.04	0.64–1.69
Number of friends/relatives who permit smoking at home (<half versus ≥half)	2.65	1.60–4.20
Current smoker	0.46	0.23–0.93

**Table 3:** Reasons for establishing a smoke-free home comparing those with a complete smoke-free home policy with those without a complete policy (%).

Characteristic	Total *N* = 500	Complete policy *n* = 176	No or partial policy *n* = 324	*P *
Health concerns^a^ score, mean ± SD	2.74 ± 0.64	2.83 ± 0.54	2.69 ± 0.65	0.045
To protect your family from the harmful effects of secondhand smoke, %	95.2	95.5	95.1	0.841
To avoid being bothered by tobacco smoke, %	88.1	93.6	85.1	0.005
To show that you care about the health of people you live with, %	90.7	93.7	89.1	0.090
Children concerns^b^ score, mean ± SD	1.79 ± 0.47	1.88 ± 0.38	1.75 ± 0.51	0.001
To discourage children from starting to smoke, %	91.8	93.7	90.7	0.265
To keep children from getting sick and missing school, %	87.5	94.3	83.8	0.001
Quitting concerns^c^, score, mean ± SD	1.80 ± 0.55	1.84 ± 0.51	1.78 ± 0.57	0.227
To encourage yourself/smokers you live with to smoke less, %	91.2	92.6	90.4	0.413
To encourage yourself/smokers you live with to quit smoking, %	89.0	91.5	87.7	0.192
Cleanliness concerns^d^, score, mean ± SD	2.37 ± 0.92	2.54 ± 0.75	2.28 ± 0.99	0.001
To avoid unpleasant odors	88.6	91.4	87.0	0.142
To make the home easier to clean, %	84.9	92.5	80.7	<0.001
To make the home easier to sell or rent, %	63.9	70.1	60.5	0.030
Other concerns^e^, score, mean ± SD	2.38 ± 0.91	2.60 ± 0.75	2.27 ± 0.96	<0.001
To avoid annoying others, %	85.3	91.3	82.0	0.005
To reduce the chance of having a house fire, %	82.6	90.8	78.2	<0.001
To protect the health of pets, %	62.1	73.8	55.8	<0.001

^a^Health concerns = sum score of three items (0 = no, 1 = yes).

^b^Kids concerns = sum score of two items (0 = no, 1 = yes).

^c^Quit concerns = sum score of two items (0 = no, 1 = yes).

^d^Cleanliness concerns = sum score of three items (0 = no, 1 = yes).

^e^Other concerns = sum score of three items (0 = no, 1 = yes).

**Table 4:** Places where smoking is allowed among participants with a partial policy versus those with no policy (%).

Characteristic	Total *N* = 324	Partial smoke-free policy *n* = 182	No smoke-free policy *n* = 142	*P *
Family/living room	64.2	60.9	68.8	<0.001
Kitchen	46.1	39.8	54.3	<0.001
Bathroom(s)	33.8	23.3	47.4	<0.001
Your bedroom	23.5	8.3	44.2	<0.001
Other adult's bedroom(s)	22.2	11.2	37.2	<0.001
Children's bedroom(s)	17.1	9.0	28.1	<0.001
